# Potential application of cryobiopsy for histo-molecular characterization of mediastinal lymph nodes in patients with thoracic malignancies: a case presentation series and implications for future developments

**DOI:** 10.1186/s12890-021-01814-x

**Published:** 2022-01-08

**Authors:** Carlo Genova, Elena Tagliabue, Marco Mora, Teresita Aloè, Mariella Dono, Sandra Salvi, Lodovica Zullo, Emanuela Barisione

**Affiliations:** 1 UOC Clinica Di Oncologia Medica, IRCCS Ospedale Policlinico San Martino, Genova, Italy; 2 Dipartimento Di Medicina Interna E Specialità Mediche (DiMI), Facoltà Di Medicina E Chirurgia, Università Degli Studi Di Genova, Genova, Italy; 3 UOC Pneumologia Interventistica, IRCCS Ospedale Policlinico San Martino, Genova, Italy; 4 UOC Anatomia Patologica, IRCCS Ospedale Policlinico San Martino, Genova, Italy; 5 UO Diagnostica Molecolare, IRCCS Ospedale Policlinico San Martino, Genova, Italy; 6 UOC Oncologia Medica 2, IRCCS Ospedale Policlinico San Martino, Genova, Italy

**Keywords:** Cryobiopsy, EBUS TBNA, Lung cancer, Lymph node, Mediastinum, Case report, Case presentation

## Abstract

**Background:**

The management of non-small cell lung cancer (NSCLC) has become increasingly complex due to the evolution of personalized medicine approaches. Such approaches are characterized by the necessity of adequate tumor samples; hence, improved biopsy techniques are needed. Transbronchial lung cryobiopsy is a novel endoscopic procedure designed to collect peripheral pulmonary tissue, and it is currently employed in interstitial lung diseases. The use of this technique in oncology might result in improved mediastinum staging and molecular characterizations; however, available data involving the use of a cryoprobe on mediastinal lymph nodes are still limited.

**Case presentation:**

Here we present a series of five consecutive patients who underwent endoscopic assessment of mediastinal lymph nodes for oncologic reasons. All patients were subjected both to endobronchial ultrasound-guided transbronchial needle aspiration (EBUS TBNA) and cryobiopsy of mediastinal lymph nodes during the same procedure, and no complications were observed. In three of the reported cases, both cryobiopsy and cell block from EBUS TBNA were positive, while in one case cryobiopsy was not diagnostic and EBUS TBNA was negative; moreover, one case showed discordance between the procedures, as cryobiopsy was negative and cell block obtained from multiple stations was diagnostic for small cell lung cancer. In one case involving a patient treated for lymphoma, cryobiopsy provided more complete histologic characterization, and in another case involving a patient affected by NSCLC cryobiopsy provided more material for molecular analyses.

**Conclusion:**

This case presentation series suggests that cryobiopsy, which has been generally used on peripheral lung lesions so far, is a feasible and safe approach for diagnosis and staging of mediastinal lymph nodal involvement, especially when station 7 is involved. Compared to EBUS TBNA, cryobiopsy might provide more adequate histological samples, with a possible impact on molecular characterizations and, therefore, therapeutic decisions. However, the learning curve of the procedure has not to be understated and optimal protocols for implementing this technique are needed. In our opinion, further studies designed to integrate the routine use of cryobiopsy in current practice for solid and eventually hematologic tumors with mediastinal lymph node involvement are warranted.

**Supplementary Information:**

The online version contains supplementary material available at 10.1186/s12890-021-01814-x.

## Background

The management of thoracic malignancies, and more specifically lung cancer, has become increasingly complex throughout the last decade; for instance, proper molecular characterization of tissue samples is now pivotal in the therapeutic approach to non-small cell lung cancer (NSCLC). The notion that a correct histological characterization is needed for the most appropriate chemotherapy regimen is widely acknowledged, as some agent are more or less effective according to the histologic sub-type, such as pemetrexed, which is employed only in non-squamous NSCLC [1]. Furthermore, additional analyses are currently considered mandatory for the management of advanced NSCLC. On one side, the expression of the ligand of programmed death protein 1 (PD-L1) is needed for determining the sensitivity to immune checkpoint blockade [2]; on the other side, molecular alterations of an increasing number of potential oncogenic drivers must be assessed in order to identify those patients who are potential candidates for targeted therapy [3].

Notably, collecting adequate tumor samples for diagnosis and molecular analyses might be difficult, especially when the only accessible source of material is represented by mediastinal lymph nodes, which are usually approached by endobronchial ultrasound-guided transbronchial needle aspiration (EBUS TBNA). This procedure is also used for mediastinal staging, with high impact on therapeutic decisions when lymph nodal involvement is suspected. Indeed, lymph nodal staging might result critical in multidisciplinary decisions for locally advanced NSCLC patients, who might be selected for neoadjuvant approaches or chemo-radiation, based on lymph nodal findings. Unfortunately, EBUS TBNA is occasionally hampered by limited or inadequate samples, thus requiring re-biopsies or additional diagnostic procedures such as mediastinoscopy when probability of malignity remains high [4]. While mediastinal lymph nodal characterization is critical in lung cancer, the utility of invasive mediastinal assessment is not limited to this disease, as other malignancies with thoracic involvement, including also lymphomas, might require this approach.

Cryobiopsy is an endoscopic technique mostly used in the diagnostic approach to interstitial lung disease, based on rapid cooling, crystallization, and subsequent collection of tissue. Since cryobiopsy allows to retrieve more material than EBUS TBNA, its use might result in more precise diagnosis, better lymph nodal staging, and wider molecular characterization.

Here we present a series of five consecutive patients who underwent both cryobiopsy and EBUS TBNA for diagnostic and staging purposes during the work-up for thoracic malignancies at our Institution. The clinical characteristics of the patients and the findings at bronchoscopy with EBUS TBNA and cryobiopsy are reported in Table 1.

**Table 1:** Characteristics of the patients who underwent cryobiopsy of mediastinal lymph nodes within our Institution and findings at cryobiopsy and trans-bronchial needle aspiration (including cell block). All the patients were male. No post-procedural complications were observed

Patient	Age (years)	Smoking status	Reason for bronchoscopy	Disease stage at the time of bronchoscopy (if applicable)	Date of bronchoscopy	Target lymph node for cryobiopsy	Target lymph node for trans-bronchial needle aspiration	Outcome of cryobiopsy	Outcome of trans-bronchial needle aspiration / cell block	Concordance or discordance between cryobiopsy and cell block
Case 1	53	Never smoker	Characterization of progressive lymphoma	IV	July 7th, 2020	Station 7	Station 7	Positive for diffuse large B cell lymphoma	Positive for lymphoma cells	Concordance
Case 2	73	Former smoker	Diagnostic suspect of sarcoidosis	Not applicable	July 17th, 2020	Station 7	Station 4R; station 7	Negative for lymphatic elements (inadequate)	Negative for sarcoidosis or lymphoproliferative disorders	Not applicable*
Case 3	51	Former smoker	Characterization of lung cancer	IV	March 5th, 2021	Station 7	Station 4L; station 7	Positive for squamous cell lung cancer	Positive for exiguous amount of non-small cell lung cancer (possibly of squamous histology)	Concordance
Case 4	66	Current smoker	Mediastinal staging for squamous cell lung cancer	III	April 14th, 2021	Station 7	Station 4R; station 7	Negative for neoplastic cells	Negative for neoplastic cells	Concordance
Case 5	77	Current smoker	Characterization of mediastinal lymph nodes	IV	May 20th, 2021	Station 10R	stations 4R, 7, 10R and 11R	Negative for neoplastic cells	Positive for small cell lung cancer	Discordance

*In this case, concordance between cryobiopsy and EBUS TBNA was not ruled out because cryobiopsy was considered not diagnostic

## Case presentation

### Patient selection and procedure

The following case presentation series includes five consecutive patients selected with the following criteria:

1. Proven or suspected malignancy, either of solid or hematologic origin.
2. Mediastinal lymph nodes with greater diameter ≥ 2 cm.
3. Necessity to undergo endoscopic mediastinal assessment for diagnosis, staging or molecular characterization, according to current practice.
4. Willingness to undergo cryobiopsy in addition to EBUS TBNA.

The eligible patients underwent EBUS with the BF-UC190F® bronchoscope (Olympus Medical Systems) under deep sedation with propofol and midazolam after signing informed consent. The procedure was performed by a pulmonologist with multi-year experience in endoscopic procedures, developed in a department with high number of yearly EBUS TBNA. After identification of suitable lymph nodes at EBUS, the pulmonologist performed TBNA with 19-gauge needle in the stations of interest (3 samplings for each station). Subsequently, the operator inserted the 1.1 mm cryoprobe (Erbe cryo®) in one station of interest. After freezing for 4 s, the operator extracted *in toto* the bronchoscope with the frozen sample attached to the tip of the cryoprobe, and the sample was thawed with saline solution. Cryosampling was performed twice. Each patient was monitored for any complication and subsequently discharged from the hospital on the same day of the procedure.

### Case 1

Patient D.M., male, aged 53, has been affected by grade 3A follicular non-Hodgkin’s lymphoma since January 2018. Since February 2018, the patient received multiple lines of chemotherapy, as it follows: R-CHOP (rituximab plus cyclophosphamide, doxorubicin, vincristine, and prednisone), followed by maintenance with rituximab alone; DHAOX (rituximab, dexamethasone, high-dose cytarabine, and oxaliplatin) followed by CD-34 apheresis and autologous cell transplant. In June 2020, a fluorodeoxyglucose positron emission tomography (FDG-PET) scan showed disease progression at multiple lymph nodal stations: 8R, 4R and 7 (SUV max 32,5), 10R and 11R (SUV max 32.5).

The patient underwent EBUS on July 7th, 2020, with evidence of dimensionally increased lymph node in station 7. EBUS TNBA smear showed small lymphocytes in the absence of atypical cellular elements; the cell block obtained from EBUS TBNA on station 7 revealed an immunological finding consistent with type B lymphoproliferative neoplasia; cryobiopsy on lymph node station 7 showed high expression of CD20 and rare CD3 elements. No complications were reported after the procedure. The cryobiopsy sample was compatible with disease evolution to diffuse large B-cell lymphoma. Based on these findings, the patient received rituximab plus bendamustine and was subsequently treated with infusion of chimeric antigen receptor-T (CAR-T) cells.

### Case 2

Patient G.M., male, aged 73, was admitted to our Institution due to persistent fever and chills, characterized by spontaneous resolution and associated with sweating. In February 2020, the patient underwent FDG-PET scan, which showed hilar-mediastinal lymphadenopathies in the following sites: 4R (SUV max 3.4), 10R (SUV max 6.3), 10L (SUV max 6.3) and 7 (SUV max 5.2).

During the diagnostic work-up, elevated angiotensin converting enzyme (ACE) was detected (> 120 U/L), leading to differential diagnosis between sarcoidosis and a lymphoproliferative disorder.

Based on these findings, an EBUS was performed on July 17th, 2020. At ultrasound, dimensionally increased lymph nodes were detected at station 4L, 4R and 7. Broncho-aspiration revealed alveolar macrophages capturing anthracotic pigment. The EBUS TBNA smears of stations 4L, 4R and 7 were negative for neoplastic elements; cell block was obtained from EBUS at stations 4R and station 7, and anthracotic histiocytes were detected in station 7. Cryobiopsy performed on station 7 was negative for neoplastic or lymph nodal elements, and hence the procedure was considered non-diagnostic due to inadequate sample. No complications were reported after the procedure.

In September 2020, a follow-up FDG-PET scan revealed persistent lymph node uptake at paratracheal site, tracheobronchial angle, right lung hilum and subcarinal station. Based on persistent fluorodeoxyglucose uptake, a mediastinoscopy was performed with confirmation of anthracotic cells and sinus histiocytes at station 2R and 4R in the absence of lymphoproliferative elements or granulomas.

### Case 3

Patient V.M., male, aged 51, was affected by diffuse bilateral pulmonary fibrosis due to systemic sclerosis since 2000. In February 2021, due to worsened cough, the patient underwent a chest high resolution (HR) computerized tomography (CT) scan, which showed a nodule (max diameter: 27 mm) at lower right lobe, lymphadenopathies in station 7 (40 × 25 mm), station 2R (26 × 27 mm) and station 10R (25 × 28 mm), as well as osteo-structural alterations at right III, IV and V ribs. An FDG-PET scan confirmed metabolic activity of the nodule in the lower right lobe (SUV max 6.5), III right rib (SUV max 3.5), right sacral wing (SUV max 11.6), lymph node stations 3 (SUV max 7.2) and station 7 (SUV max 8.2).

On March 5th, 2021, the patient underwent EBUS and ultrasound revealed increased lymph node size at 4L, 4R and 7. We collected smears on stations 4L and 7 and a cryobiopsy on station 7. The histologic report of the EBUS TBNA (March 13th, 2021) on station 4L e 7 showed an exiguous amount of NSCLC cells possibly deriving from squamous cell lung cancer; the cryobiopsy on station 7 was diagnostic for squamous cell lung cancer and included enough material for comprehensive molecular characterizations. No complications were reported after the procedure.

Although the histological report of the cryobiopsy was compatible with squamous histology, we nonetheless performed molecular analyses for detection of actionable oncogenic drivers, due to the young age and limited smoking history. The molecular report (March 25th, 2021), however, was negative for currently actionable drivers and we identified low expression of programmed death protein 1 ligand (PD-L1). Subsequently, the patient underwent first-line chemotherapy with carboplatin-paclitaxel until disease progression and ultimately death.

### Case 4

Patient M.I., male, aged 66, underwent a chest CT scan on December 29th, 2020, during hospitalisation for Sars-CoV-2 infection. The exam showed an incidental finding of bilateral pleural effusion and right lower lobe lesion (52 × 44 mm). Thus, the patient underwent lung needle biopsy on March 3rd, 2021, with the histopathological diagnosis of a squamous cell carcinoma of the lung (PD-L1 55–60%). Subsequently, the patient underwent radiological staging with CT scan of brain, chest and abdomen and FDG-PET, which confirmed the presence of a single lesion in the right lower lobe and detected mediastinal lymphadenopathies (up to 45 × 20 mm; SUV max up to 4.0) in stations 4R and 7. No distant metastases were detected.

On April 14th, 2021, the patient underwent EBUS for the purpose of mediastinal staging. At ultrasound, increased lymph nodes were detected at station 4L, 4R and 7. Cytoaspiration from stations 4L, 4R and 7 and cell block from stations 4R and 7 were performed. Both cytoaspiration and cell block were negative for neoplastic cells. Cryobiopsy was performed at station 7, with no evidence of tumour cells. No complications were reported after the procedure.

The patient was not candidate for surgery due to impaired pulmonary function. Furthermore, imaging and metabolic findings at FDG-PET were inconsistent with the histologic report of bronchoscopy, and mediastinoscopy could not be proposed because of the aforementioned pulmonary function, limiting the possibility of definitive confirmation or exclusion of lymph nodal involvement, as proposed by ESMO guidelines [5].

Based on the persistent suspect of lymph nodal involvement, the patient was considered affected by stage III, unresectable squamous NSCLC and thus he underwent sequential chemo-radiation, including platinum-based chemotherapy for three cycles, followed by thoracic irradiation with inclusion of stations 4R, 4L, and 7. After completing chemo-radiation, before starting any maintenance treatment, the patient underwent response evaluation with a chest-abdomen CT-scan, which showed disease progression due to the appearance of new pulmonary lesions, distant from the radiation field. Hence, the patient started receiving pembrolizumab as single agent for metastatic disease.

### Case 5

Patient B.G, male, aged 77, was admitted to our Institution on April 21th, 2021, for accidental trauma and persistent lumbosacral pain. After admission, he underwent lumbosacral column magnetic resonance imaging (MRI) with the accidental finding of altered signals at multiple vertebral somas (D12, L2, L4). Therefore, an FDG-PET scan was performed on May 5th, 2021, showing multiple metabolically active mediastinal lymph nodes and bone lesions at D11, D12, L2 and L4 (SUV max 4.7) and in pelvis (SUV max 3.1). In order to obtain a histopathological diagnosis, the patient underwent EBUS TBNA on May 20th, 2021. The ultrasound probe revealed lymph node stations with increased volume and dishomogeneous structure at stations 4R, 7, 10R and 11R. Smears from EBUS TBNA were negative; however, cell block obtained from station 10R and station 7 was diagnostic for small cell lung cancer (SCLC). Notably, cryobiopsy performed on station 10R did not demonstrate the presence of tumour cells, in contrast with cell block. No complications were reported after the procedure.

Based on the histology and disease stage, the patient received first-line treatment with carboplatin, etoposide and atezolizumab for 4 cycles followed by maintenance therapy with atezolizumab, which was ongoing with disease control at the last follow-up in October 2021.

## Discussion and conclusion

The proper characterization of mediastinal lymph nodes during the work-up for thoracic malignancies or lymphoproliferative disorders is currently a critical element in terms of diagnosis and staging. On one hand, mediastinal lymph nodes might represent the most easily accessible disease site in many cases of lung cancer, including both NSCLC and SCLC; on the other hand, mediastinal lymph nodes might represent the only site of disease, as it may be observed in lymphoproliferative disorders. Hence, the collection of adequate samples from mediastinal lymph nodes might be mandatory for the correct histologic diagnosis and, in the case of some solid tumors, for a comprehensive molecular characterization, which is not required for diagnosis, but is critical for therapeutic decisions.

Indeed, with specific regard to NSCLC, the number of potentially actionable oncogenic drivers is constantly increasing. While the first acknowledged driver was the epidermal growth factor receptor (*EGFR*), novel biomarkers of therapeutic interest are emerging, thus requiring increasing quantity of tumor tissue for testing multiple biomarkers at once. This is especially relevant considering the role that next generation sequencing (NGS) is acquiring in current clinical practice. More specifically, some potential oncogenic drivers such as *EGFR*, anaplastic lymphoma kinase (*ALK*), c-Ros oncogene 1 (*ROS-1*), and V-raf murine sarcoma viral oncogene homolog B (*BRAF*) are considered mandatory targets for molecular testing. However, current guidelines for the management of NSCLC agree that all the patients affected by non-squamous NSCLC and those affected by squamous NSCLC but with light/no smoking history should be considered for comprehensive molecular panels, not limited to the aforementioned genes. Indeed, the increasing availability of effective molecular therapies directed towards novel targets, such as Mesenchymal–Epithelial Transition factor (*MET*), Rearranged during Transfection (*RET*), Neurotrophic Tyrosine Kinase (*NTRK*), and Kirsten Rat Sarcoma viral oncogene (*KRAS*), and the evolution of molecular sequencing technologies demand for extensive analyses. In this context, the tissue collected at diagnosis needs to be adequate both in terms of quantity and quality, in order to prevent repeated procedures [6].

Furthermore, recent advances in the management of locally advanced NSCLC associated with the availability of maintenance with durvalumab after chemo-radiation, as well as chemo-immunotherapy combinations in the neoadjuvant setting, have led to renewed interest in the approach to stage III NSCLC. In this scenario, adequate mediastinal lymph nodal staging is critical in determining whether a patient with stage III NSCLC due to mediastinal, non-bulky, lymph nodal involvement, should be considered potentially resectable after neoadjuvant chemotherapy or whether a chemo-radiation approach, eventually followed by maintenance with the immune checkpoint inhibitor durvalumab, should be considered. Noteworthy, in the latter case, adequate tissue for PD-L1 expression analysis might be required, according to the current registration of durvalumab in Europe [6].

While EBUS TBNA has proven extremely valuable for endoscopic diagnosis and mediastinal staging in NSCLC and other thoracic malignancies, it occasionally provides a limited sample, inadequate for comprehensive analyses or even for diagnostic purposes. Furthermore, when lymphoproliferative disorders are involved, the lack of histologic information severely limits the usefulness of EBUS TBNA. In solid tumors, the collection of limited samples might result troublesome, especially when the only accessible lesions are mediastinal lymph nodes or when the correct identification of lymph nodal metastases is required to define the oncologic approach for each patient, such as defining the extension of a locally advanced NSCLC for inclusion in chemo-radiation regimens. Hence, novel diagnostic techniques for appropriate lymph nodal sampling are warranted.

Cryobiopsy is a relatively novel endoscopic technique designed to approach the region of interest by freezing and collecting a more adequate amount of tissue with negligible complications [2, 3]. This procedure has recently become a diagnostic cornerstone for interstitial lung disease [9]. Retrospective data on a small subset of cancer patients suggest that cryobiopsy performed on peripheral lesions might provide more adequate samples for NGS compared to EBUS TBNA on mediastinal stations, allowing to analyze more genes (p = 0.024) [10]. Nonetheless, its use in the transbronchial approach to mediastinal lymph nodes in cancer management is still limited, possibly due to the need of specific training for developing adequate skill in both endoscopy and cryobiopsy. To date, another case series, including 4 patients, suggests the feasibility and safety of cryobiopsy for mediastinal lymph nodes, and its consistency with EBUS TBNA [11].

**Fig. 1:**
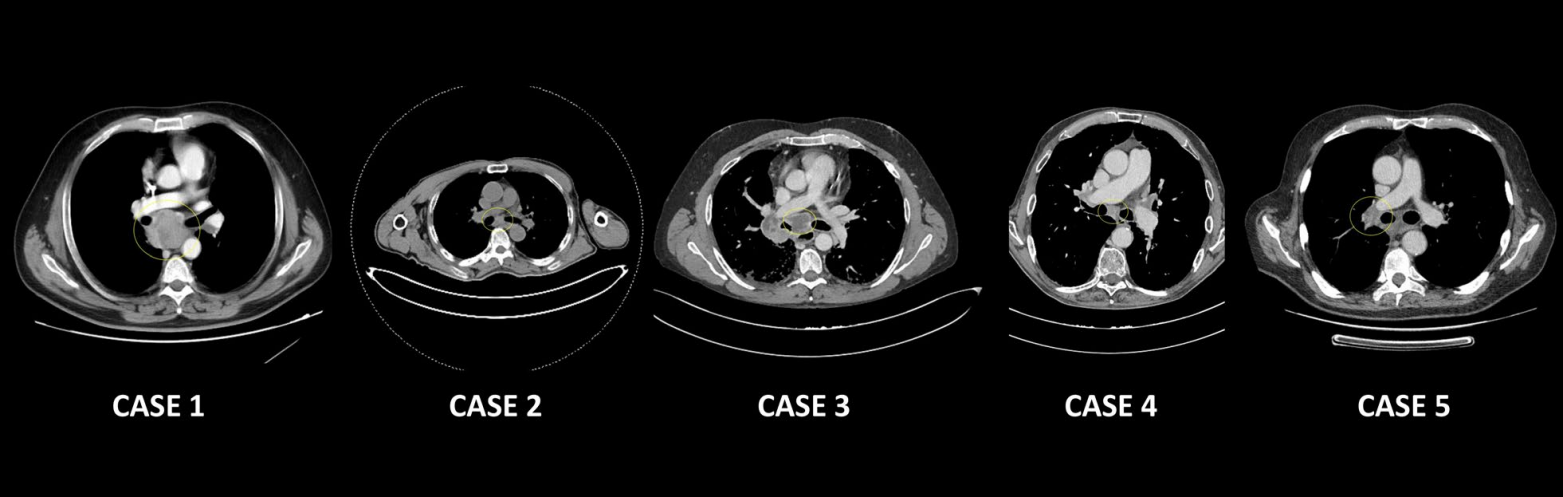
Relevant scans of the lymph nodal stations approached with cryobiopsy in each case presentation

**Fig. 2:**
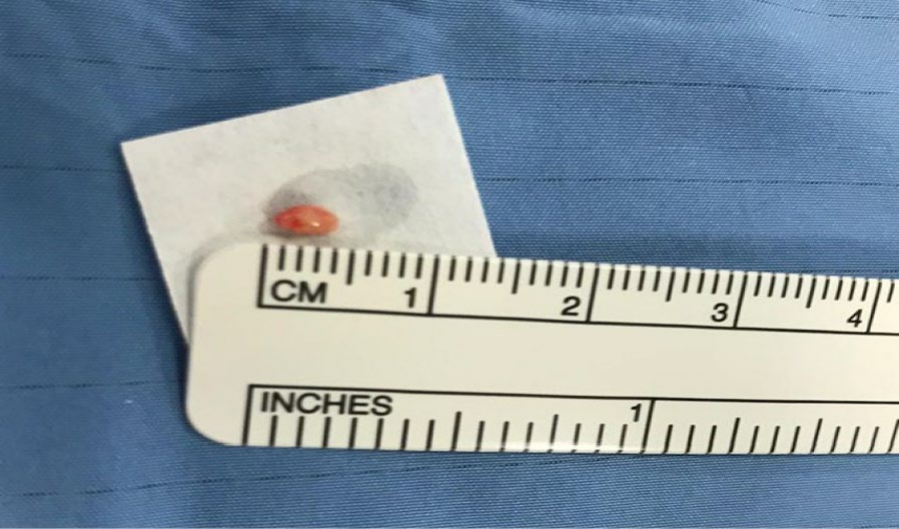
Bioptic sample collected through cryobiopsy

Our case series includes patients for which endoscopic mediastinal assessment was crucial for diagnostic or staging reasons, and who underwent both EBUS TBNA and cryobiopsy during the same endoscopic procedure. The lymph nodes approached by cryobiopsy in each case are reported in Fig. 1, while an example of cryobiopsy sample from *case 3* is reported in Fig. 2 and microscopy fields at original magnification 20 × are reported in Additional file 1: figures A, B, C. In the first case, the patient was affected by lymphoma and endoscopic approach was required to verify a shift towards a more aggressive histology and hence modulate antineoplastic treatment. In this case, EBUS TBNA was itself positive for lymphoma cells, but cryobiopsy allowed a more precise characterization, which demonstrated the development of a diffuse large B cell lymphoma. In the second case, the patient underwent bronchoscopy to rule out a diagnostic suspect of lymphoproliferative disorder or sarcoidosis. EBUS TBNA was negative, while cryobiopsy was not diagnostic; a subsequent mediastinoscopy, performed for persistence of diagnostic suspect at metabolic imaging, confirmed the endoscopic findings.

In the third case, bronchoscopy was performed in order to provide molecular characterization of NSCLC; in this case, EBUS TBNA provided an exiguous sample, whereas cryobiopsy provided enough tissue for a precise histologic diagnosis and subsequent molecular characterization.

In the fourth case, bronchoscopy was required for mediastinum staging and both cryobiopsy and EBUS TBNA were consistently negative; nonetheless, the investigated stations were included in the radiation field during sequential chemo-radiation. Notably, disease progression occurred in different sites, and no lymph nodal progression was observed.

In the fifth case, cryobiopsy and cell block were not consistent, and the latter provided diagnosis of SCLC, while cryobiopsy was negative. In this case, we observed that cell block was obtained from multiple mediastinal lymph node, while cryobiopsy was performed only on one station. Furthermore, this is the only case where 10R station was approached by cryobiopsy, while the other cases involved station 7. This finding suggests that the learning curve for cryobiopsy may vary among different stations, and sub-carinal lymph nodes represent a more easily approachable target for anatomic reasons.

Based on the global observations of our case presentation series, we believe cryobiopsy is a safe and feasible diagnostic and staging approach to mediastinal lymph nodal sampling in thoracic malignancies, although the procedure should be reserved to experienced operators in order to be effective.

At present time, while the required expertise is being developed, one possible approach might be based on the conjunction of cryobiopsy and EBUS TBNA within the same procedure. In first place, adding the cryobiopsy to standard EBUS TBNA does not appear to increase discomfort or risks related to the procedure, at least based on our experience and the recent series published by Gonuguntla et al. [11]. In second place, the simultaneous use of cryobiopsy and EBUS TBNA might result in more informative sample collection, as cryobiopsy allows to obtain actual histologic samples, potentially more informative on tumor architecture and with more material for comprehensive molecular analysis than cytologic samples, while at the same time EBUS TBNA can be easily performed on multiple lymph nodal stations at the same time, allowing to approach stations which might be difficult to access with cryobiopsy. Finally, the concordance between EBUS TBNA and cryobiopsy might allow to increase the accuracy of the whole procedure by limiting the risk of false negatives.

In conclusion, cryobiopsy is a promising technique to explore mediastinal lymph node stations, although at present time, while expertise on the procedure and proper lymph node selection is being acquired, the most effective approach appears to include both EBUS TBNA and cryobiopsy. In our opinion, prospective studies designed to integrate the routine use of cryobiopsy in current practice for thoracic malignancies with mediastinal lymph node involvement are highly warranted, as they might improve the procedure and ultimately result in increasing personalization of antineoplastic treatments by providing more adequate tumors samples.

## Supplementary Information

**Additional file 1.** Microscopy fields from the case 3 cryobiopsy.

## Data Availability

No additional support data (including raw data) have been generated for the development of this manuscript, due to its nature of case report. Full data, including complete CT scans and microscope images, are held by Prof. Carlo Genova (carlo.genova@hsanmartino.it) and available upon request.

## Abbreviations

ALK: Anaplastic lymphoma kinase
BRAF: V-raf murine sarcoma viral oncogene homolog B
CT-scan: Computerized tomography scan
EBUS TBNA: Endobronchial ultrasound-guided transbronchial needle aspiration
EGFR: Epidermal growth factor receptor
FDG-PET: 18-Fluorodeoxyglucose positron emission tomography
FISH: Fluorescence in situ hybridization
IHC: Immunohistochemistry
KRAS: Kirsten rat sarcoma viral oncogene
MET: Mesenchymal–epithelial transition factor
NGS: Next generation sequencing
NSCLC: Non-small cell lung cancer
NTRK: Neurotrophic tyrosine kinase
PD-L1: Programmed death protein ligand 1
RET: Rearranged during transfection
ROS-1: C-Ros oncogene 1
SCLC: Small cell lung cancer
