# Supplementary material for: Potential application of cryobiopsy for histo-molecular characterization of mediastinal lymph nodes in patients with thoracic malignancies: a case presentation series and implications for future developments
Source: BMC Pulm Med. 2022 Jan 8;22:5. doi: 10.1186/s12890-021-01814-x (PMC8741535; doi:10.1186/s12890-021-01814-x)

**SUPPLEMENTARY FIGURES**

Microscopy fields from the case 3 cryobiopsy.

1. hematoxylin and eosin staining; B) p40 staining; C) TTF-1 negative staining.

The images were collected through Leica DMD108 digital microimaging network and were reported avoiding threshold manipulation, expansion or contraction of signal ranges and the altering of high signals.

The figures were acquired at original magnification 10x.

**A**


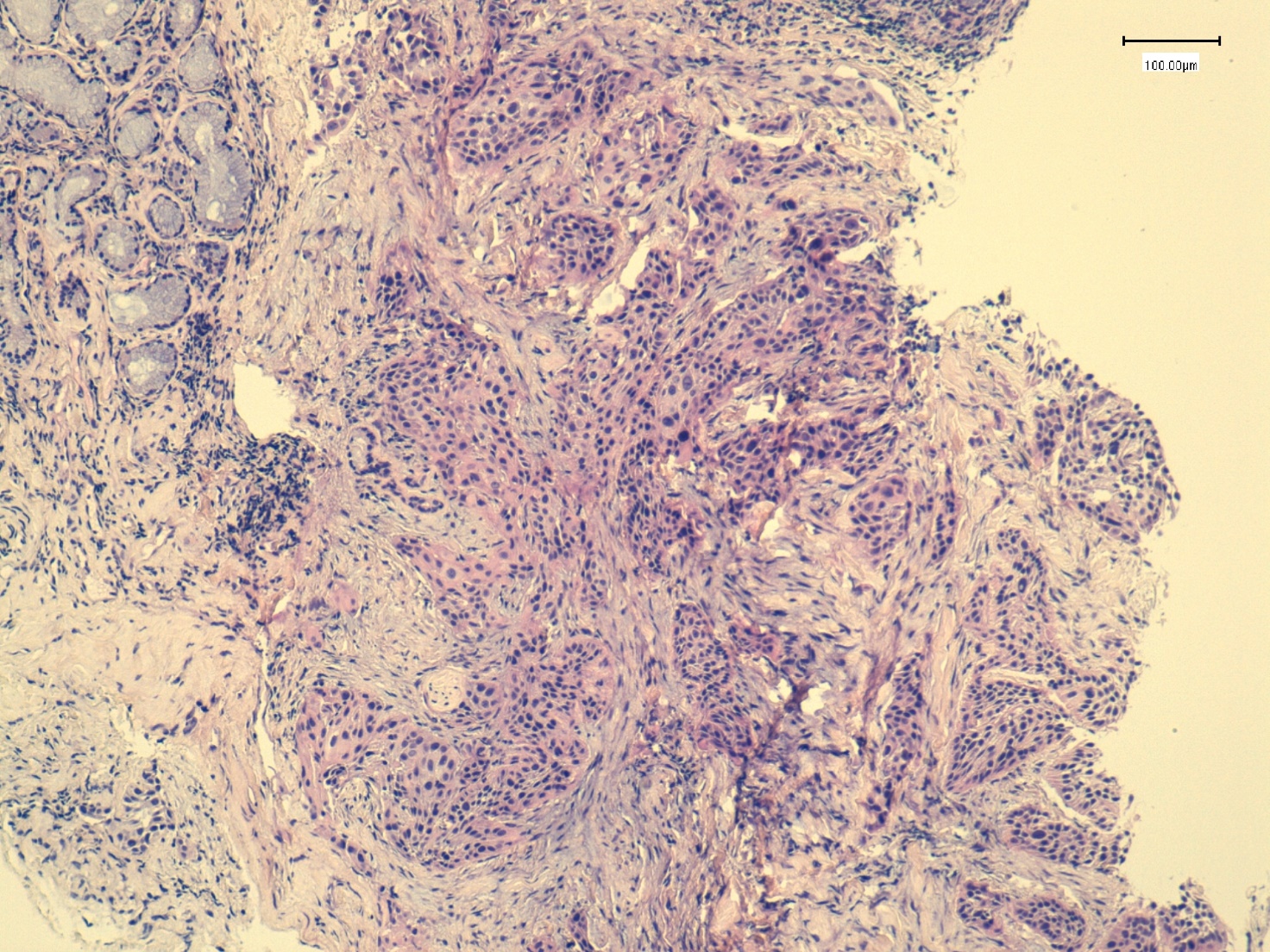


**B**


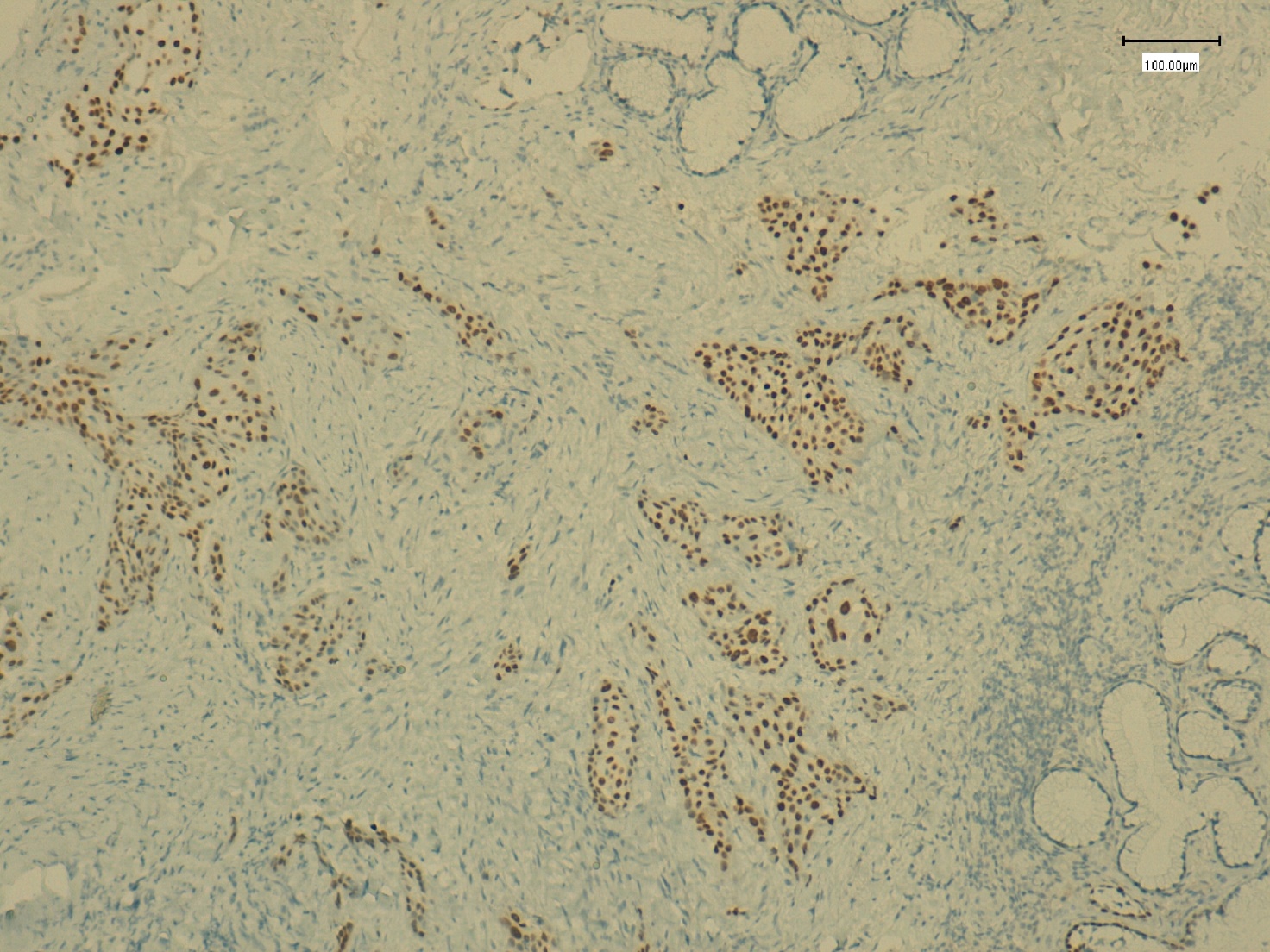


**C**


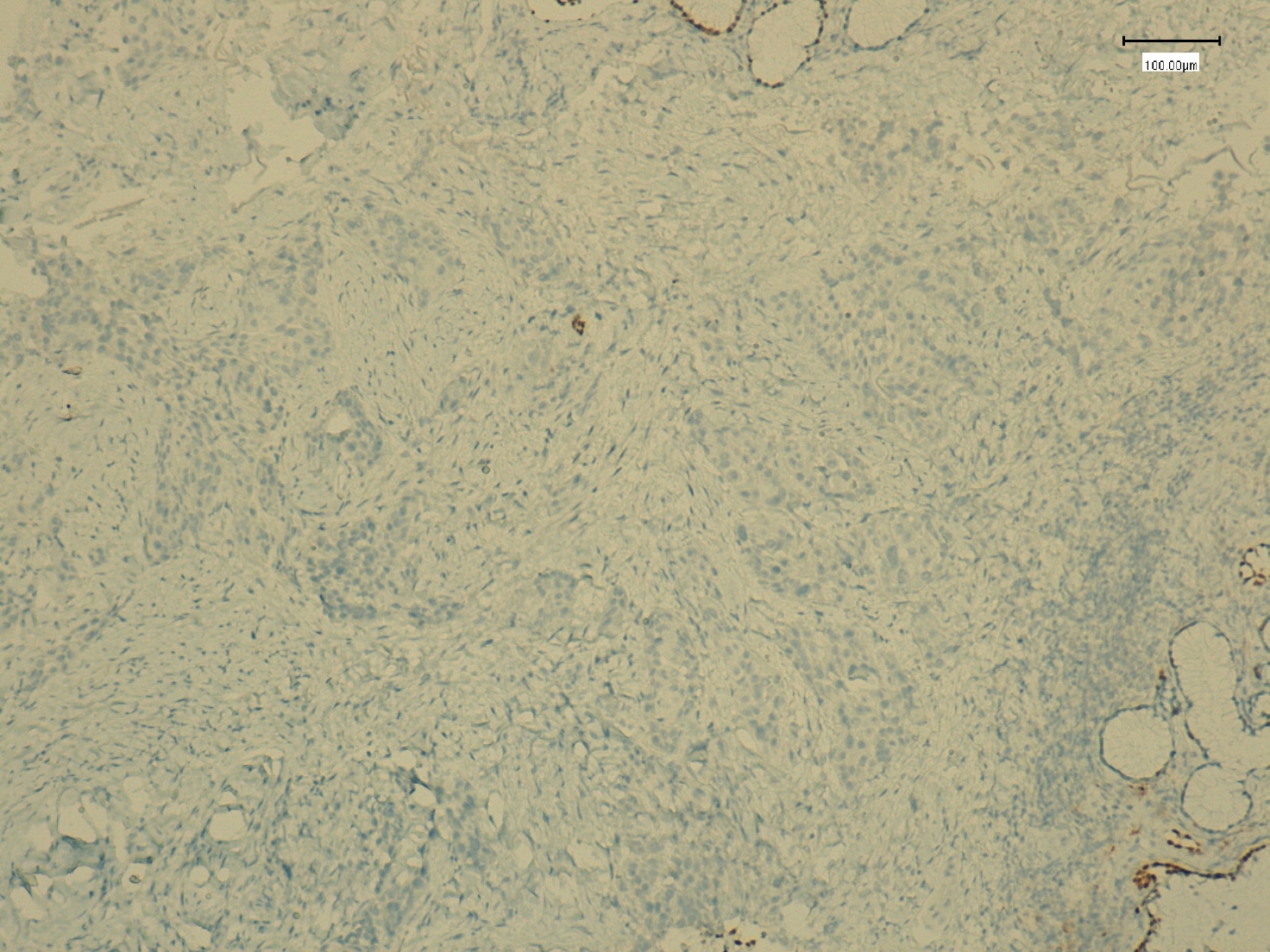

Supplement: Supplementary file 1 — Additional file 1. Microscopy fields from the case 3 cryobiopsy. [file 12890_2021_1814_MOESM1_ESM.docx]
